# Barriers and facilitators of using dementia community support services provided by an Alzheimers organisation: Perceptions of informal caregivers receiving services

**DOI:** 10.1111/hsc.13796

**Published:** 2022-03-18

**Authors:** Kirsten J. Robertson, Maree Thyne, Ivanna Matheson

**Affiliations:** ^1^ Department of Marketing University of Otago Dunedin New Zealand; ^2^ 2495 Department of Marketing University of Otago Dunedin New Zealand

**Keywords:** Alzheimers, Alzheimers association, dementia, barriers, facilitators, informal caregiver's needs, service use

## Abstract

Known barriers prevent informal caregivers of a person with dementia using community services; however, there is a dearth of knowledge on how organisations can overcome these barriers. This study examined caregivers’ perceptions of the barriers and facilitators of service use with regards to their membership to one Alzheimers organisation and their recommendations for improvements. In‐depth interviews were conducted with 19 informal caregivers. Thematic analysis revealed personal and organisational barriers to service use, and associated recommendations. Six recommendations were made for dementia service organisations: (a) be proactive and arrange regular scheduled meetings with clients; (b) utilise consistent, trusting, empathic support personnel who can build strong relationships with clients; (c) provide support groups; (d) tailor support; (e) ensure expert knowledge and numerous channels of information delivery to clients, the general public and health professionals and (f) actively promote the organisation and services offered. This study provides novel insights into how a community organisation can overcome client barriers to service use. In addition, the study reveals caregivers perceived value of an Alzheimers organisation, argued to be an essential service, but until now clients’ perceptions of the value received have not been explored.

**What is known about this topic**

- There is a trend for low utilisation of community services by the persons with dementia and their caregivers.
- Barriers to service use are well known.

**What the paper adds**

- Empathetic staff, providing support groups, proactively contacting clients, and providing expert information and education overcame barriers to service use.
- It is important to normalise the difficult emotions caregivers’ experience.
- Without the organisation being proactive, caregivers continue to ‘try and cope’ on their own.

## 1 INTRODUCTION

Dementia incorporates many types of neurodegenerative brain syndromes, including Alzheimers disease, and is a major health issue and public health priority (World Health Organization, 2012). The World Health Organization (2017) estimates that 47 million people worldwide have dementia, potentially tripling by 2050. Most OECD countries aim to support the person with dementia (PWD) to live at home for as long as practical (Organisation for Economic Co‐operation & Development, 2015). Care is primarily provided by informal (family) caregivers (Brodaty & Donkin, 2009) who require adequate support, without which they tend to experience burden (Pinquart & Sörensen, 2003). Support is typically provided by public healthcare and dementia care community support services like Alzheimers Associations (Ploeg et al., 2009). However, due to a wide range of barriers (see e.g. Moholt et al., 2020), caregivers often do not use available services (Weber et al., 2011). Although barriers to service use are well known, there is a dearth of knowledge on how organisations may overcome these barriers. Using insight provided by informal caregivers, the present study asked members of one Alzheimers organisation in New Zealand to state their perceptions of barriers and facilitators to service use and recommendations on how the organisation could overcome barriers.

Alzheimers Associations have been established worldwide (Wortmann, 2012), with associations in 123 countries all with a shared aim of providing support to carers and the PWD (Alzheimer's Disease International – Member Associations, 2021). In New Zealand, Alzheimers New Zealand has 14 different regional societies across the country (Alzheimer’s New Zealand, 2021). Services to carers and the PWD vary but can include support groups, helplines (Wortmann, 2012), emotional support and information (Brodaty & Donkin, 2009). Researchers have argued that Alzheimers Associations are crucial for linking caregivers with local support (Brodaty & Donkin, 2009), are an essential service that should be funded by national governments and that caregivers should be informed of the local Alzheimers Association at diagnosis (Georges et al., 2008). Research has found support groups (one component of the service offered by Alzheimers Associations), reduce caregiver's social isolation, improve their psychological wellbeing (Chien et al., 2011) and provide helpful advice (Lauritzen et al., 2015). However, caregivers’ perceptions per se of what facilitates their association with an Alzheimers organisation, have been unexplored and will be addressed in this study.

### 1.1 Burden on informal caregivers

Informal caregivers have been referred to as 'invisible second patients' (Brodaty & Donkin, 2009, P. 217). Caring for a PWD is associated with poor physical and psychological health (Collins & Kishita, 2019; Gilhooly et al., 2016) and without appropriate support, caregivers tend to have lower life satisfaction (Pinquart & Sörensen, 2003). Moreover, caregiver burden is associated with worsening health in the PWD and an increased likelihood of institutionalisation (Stall et al., 2019). Supporting caregivers is essential to lowering burden and delaying the admission of the PWD into an institution (Afram et al., 2014); however, support for caregivers is unsatisfactory, especially regarding emotional support (Swain, 2018).

### 1.2 Reasons behind service underutilisation

There is a significant trend for low utilisation of community services by the PWD and their caregivers (Weber et al., 2011). A substantial barrier is a lack of awareness of service providers. For instance, in Canada, where there are 10 provincial Alzheimers Associations with further local societies (Alzheimer’s Society Canada, 2021) although the Alzheimers Association was the most widely recognised source of community support, it was only mentioned by 18% of a random telephone sample of 1,152 adults over 50 years (Ploeg et al., 2009). Further research has shown that potential caregivers lack sufficient information on organisations, their services (Brodaty et al., 2005), or how to access services (Laparidou et al., 2019). For instance, a study in Europe, where there are 39 Alzheimers Associations across 35 countries (Alzheimer’s Disease International – Regions, 2021) found 59% of caregivers were uninformed of the Alzheimers Association when the PWD was diagnosed (Georges et al., 2008). Although a lack of awareness of services is clearly a barrier, other researchers argue that barriers are multifaceted (Moholt et al., 2020). Barriers are systemic of the wider healthcare system, which has been found to be confusing and impenetrable (Peel & Harding, 2014), and healthcare practitioners are perceived to lack training or knowledge in dementia care services (Laparidou et al., 2019).

In addition to barriers attributed to healthcare and support systems, other barriers to service use involve attributes, and specific needs of caregivers or the PWD themselves. Key reasons include caregivers’ perceived lack of need (Moholt et al., 2020), or the PWD not wanting/allowing help to be sought (Brodaty et al., 2005; Moholt et al., 2020; Stephan et al., 2018). Further barriers include the caregiver being housebound due to their caregiving role, caregivers dislike of untailored services (Granbo et al., 2019) and perceptions around the lack of meaningful activities for the PWD (Black et al., 2013).

Researchers have suggested strategies to overcome underutilisation of services, including the need for tailored and targeted information and education about available services and their benefits (Moholt et al., 2020; Queluz et al., 2020). However, targeting services to caregivers does not overcome under usage (Potter, 2018). Some caregivers will not ask for respite care despite needing it (Van Exel et al., 2008). Thus, providing information alone is insufficient to facilitate service use, and this could be due to emotional barriers.

Overwhelming emotions, such as a desire for privacy, fear, anxiety, (Stephan et al., 2018) and guilt (Innes et al., 2005) are further barriers to service use. Furthermore, use of services threatens caregiver's pride/agency, because caregivers perceive using services suggests they have failed (Lloyd & Stirling, 2011). One study that addressed the dearth of knowledge on how service providers can overcome barriers found having a consistent, empathic and trustworthy support person key to overcoming barriers (Stephan et al., 2018). However, Stephan et al. (2018) looked at barriers to a wide range of formal care and, in line with Queluz et al. (2020), called for future research to extend the field by focusing on specific services. Furthermore, many of the organisational recommendations in the study by Stephan et al. (2018) were drawn from the perspective of healthcare professionals. How caregivers perceive organisations overcome barriers to service utilisation remains unclear. *The current study examines caregivers experience of membership with one Alzheimers organisation (who employ constant key support workers—Community Educators), to examine the relationship between caregivers’ needs and their perceptions of the facilitators and barriers to using the services provided*.

### 1.3 New Zealand (NZ) context

In a population of 4.8 million, there are almost 70,000 people with dementia in NZ; this is estimated to increase to 170,000 by 2050 (Alzheimer’s New Zealand, 2020). Individuals in NZ are encouraged to 'age in place' (Dalziel Hon, 2001), thus, informal caregivers play a crucial role in supporting the PWD (Wong‐Cornall et al., 2017). This care is predominantly provided by partners and adult children, with most carers being women (Lapsley et al., 2020). Services for the PWD, their caregiver, and family are predominantly provided by the formal healthcare system and community organisations like the Alzheimers Association. Services provided by the Alzheimers organisation in this study include one‐to‐one support of carers and the PWD, support groups, coffee groups, and cognitive stimulation therapy. Despite two studies in NZ showing caregivers of a PWD express positive outcomes associated with using care services, uptake of services is limited (Brunton et al., 2008; Roud et al., 2006). Thus, the present study will explore caregivers of a PWD (who are members of one Alzheimers organisation) and their: (a) perceptions of service use barriers, (b) perceptions of facilitators of service use and (c) recommendations for improving the services offered by the organisation.

## 2 METHODS

### 2.1 Participants

Nineteen informal caregivers (>18 years), and a member of one Alzheimers organisation took part. Caregivers were mostly women (*n* = 15), average age of 75, and predominantly the care recipients’ spouse (*n* = 14). The average length of caregiving was 6 years (range 18 months–13 years). Convenience sampling was employed. Community Educators (who provide one‐on‐one support to carers) working for the Alzheimers organisation asked clients if they were interested in taking part in the study. Community Educators passed interested participants’ contact details onto the research team, they were then contacted about the study.

### 2.2 Design and procedure

A qualitative methodology was employed to enable an in‐depth insight into caregiver perceptions (Novais et al., 2017). Data were collected between November 2019 and February 2020. Participants were invited to take part in an open‐ended face‐to‐face interview at their home (usually lasting 1‐hr). Participants were asked to discuss their caregiving journey, knowledge of the organisation's services, the services and support they had received, their perceptions of this support, their thoughts on the facilitators and barriers to service use, and their recommendations for the organisation. The study was reviewed and approved by the University of Otago ethics committee, and all participants gave their written and informed consent.

### 2.3 Analysis

The interviews were audio recorded and then transcribed verbatim, resulting in 150 pages of single‐spaced dialogue. Thematic content analysis was conducted using Braun and Clarke's (2006) framework. Two researchers read the transcripts several times to become familiar with the data, identify emerging themes, and to develop a coding scheme. Subsequently, the two researchers independently coded the transcripts, regularly comparing coding. The codes were then sorted into overarching themes with similar meanings. Barriers, facilitators and recommendations were coded separately. Due to an overlap between barriers and recommendations, the recommendations were collapsed into themes with the barriers. Pseudonyms were used for confidentiality.

## 3 FINDINGS

Six themes were identified: caregivers’ personal barriers impacting service use; barriers of the organisation and associated recommendations; four themes around facilitators of service use, and one message was overarching (*it's different for everyone*). The themes are listed in Table 1.

**TABLE 1:** Perceived barriers and facilitators of service use and recommendations for the organisation

	Theme	Sub‐theme	Example
Perceptions of service use barriers	1. Personal barriers	Housebound	‘I was in the stage with my wife where I couldn't get out, I was literally housebound’ (P5)
		Grief	‘And I guess with my grief and because I’ve never had a break in all this time, I’m feeling like oh, I just can't do this anymore’ (P10)
		Guilt	‘you've got to stop feeling guilty about it. But then I didn't ask him to get Alzheimer's any more than he asked me to get all this wrong either’ (19)
		Privacy, pride and embarrassment	‘And I think not all people like to admit that they're struggling and don't know… it's really hard sometimes to talk about it and admit that it is really challenging and that you don't know where to go… I just had to say I don't know what to do anymore’ (P13)
		Fear of not being understood	‘People don't really understand unless they've been there, so you just tend to gloss over it, you don't really want to say much’ (P9)
		A sense of burden	‘Because sometimes you feel like a bit of a nuisance, you don't like adding to their burdens of busyness and that sort of thing’ (P11)
	2. Limited awareness and value of the organisation	Limited promotion	‘I just think people need to know what's available’ (P2)
		Limited tailored services	‘One thing that would have been good for her to, um, have had a support group of people who are in similar levels’(P15)
		Lack of proactiveness	‘He wouldn't ask for it, but he could do with the contact, he could do with that kind of thing. But the Alzheimer's society would have to be proactive and a bit bull doggy about doing that’ (P1)
		Limited education services	‘But for awareness of people in the services. Where there may be a problem… So, so greater community awareness is important’ (15)
Facilitators/service		1. Proactive regular contact	‘I got back from holiday there was a note from [the Community Educator] saying she hoped I’d enjoyed my break up North and that she'd call me to make an appointment to catch up, which she did’. (3)
		1. Empathetic	‘Very impressed with her and her empathy and understanding’ (P5)
		2. Support groups	‘And I think that you can sit there and talk about the experience with people who are going through the same thing [support group]’ (P11)
		3. Provision of information/education/referrals	‘Providing information and they do have a good range of information’ (13)

### 3.1 Overarching

The overarching message, *it's different for everyone*, demonstrated the diversity of experiences. Several caregivers noted that the experience of being a caregiver, challenges faced, and benefits received from support services, differ for everybody. Caregivers noted that everyone has different ways of managing things, that everyone copes differently, and families are different in how they react.

### 3.2 Service use barriers

#### 3.2.1 Personal barriers

##### Housebound

Many of the caregivers mentioned that the 24/7 nature of caring for a PWD meant that they were unable to leave the house: ‘I can't leave him with anybody for (even) an afternoon’ (P9). Being housebound was a barrier to service use, but also to other forms of social interaction: ‘I couldn't leave the house basically in five years, so it meant I gave up my Rotary, I gave up a lot of personal things’ (P12).

##### Grief

Caregivers also experienced grief and explained that this was different to the grief experienced when someone passes because it begins early, often as the dementia progresses, and the PWD changes: ‘I think I went through a lot of emotion watching mum change. And a lot of grieving then’ (P13). Feelings of grief resulted in seclusion, with grief preventing caregivers wanting to be around others: ‘Because with grieving…, you shun the outside world and normal life’ (P17).

##### Guilt

Guilt was linked to feeling that they were not doing enough for the PWD: ‘…that's where the guilt comes in… because you want to be able to make their life as normal as possible but to do that you take your own life’ (P13). Guilt was also a barrier to using services: ‘That's the difficult part of the guilt you feel, for leaving him behind… because you've had to put him somewhere’ (P9). Once the PWD was in care, guilt was experienced for not visiting the PWD enough: ‘I still feel guilty about not having visited mum this week’ (P13).

##### Privacy, pride and embarrassment

Pride and embarrassment prevented caregivers seeking help: *‘*It's very difficult when you're older, you know, people can be very private’ (P19). Caregivers felt that they should be able to cope: ‘you think you can do everything yourself but sometimes you can't… it's a pride thing too, you're used to dealing with them and that's your partner’ (P1). Due to their sense of privacy and feeling they should cope, caregivers expressed embarrassment at seeking help: ‘you just feel a bit humiliated asking for help. You feel like you should cope, it's your husband, your responsibility’ (P9).

##### Fear of not being understood

Caregivers expressed being secluded because they couldn't discuss their situation with others: ‘And it's certainly not something you can talk about with people’ (P13). They felt that people just wouldn't understand their situation ‘you can't talk anything about dementia to a normal person, you just can't. They just don't know… give up talking to anybody, because they don't understand’ (P11).

##### A sense of burden

Not wanting to burden the organisation also led to seclusion: ‘I just kind of coped and I thought well I don't need to annoy you’ (P10). Caregivers noted that the organisation was stretched and they only asked for help in times of desperation: ‘you're aware that they have a huge case load and they have demands from a lot of other people and they're a service that's probably spread as thin as everything else so I tended only to contact people if I was absolutely hitting the wall’ (P13).

#### 3.2.2 Limited awareness, and perceived value of the organisation

##### Limited promotion

Several caregivers mentioned they felt that people are not aware of the organisation or the services they provide: ‘I don't think that people know enough about the Alzheimers society’ (P1). A tangible suggestion made by caregivers is that the support groups should be advertised in the newspaper: ‘…there's so many who don't come to the meetings you probably don't know about it. So it should be advertised, like something in the paper.’ (P6). Another tangible suggestion offered by caregivers was services provided by the organisation should be listed in their newsletter.

##### Limited tailored services

Several caregivers noted that a lack of tailored services for the PWD was a barrier to service use: ‘I look at some of the things that are available, and I think oh my goodness… I don't know how I would cope with ‘A’ going there… because I don't think he would cope, and I don't want to see him unhappy’.(P2). Caregivers wanted individualised services: ‘One of the things I think is really important, is that each person is treated as individuals… Just enabling him to do ordinary things…’ (P17). This included more activities for the PWD: ‘I would also love to see some smaller things set up, such as an art group or dancing or sculpture. So, it doesn't feel like a therapy session, but it feels like you're going to a hobby’ (P2).

##### Lack of proactiveness

Caregivers felt that the organisation could be more proactive by anticipating needs: ‘…when we most need help that's when it's the hardest to do. People need to anticipate you wanting or needing help… even put it in your face. Even if it's not what you want’ (P17). Caregivers recommended the organisation plan regular appointments with clients: ‘Maybe they could come and visit you at a certain time once a month. That gets you through, you know it's coming up and you just have to hang on to a certain point’ (P11).

##### Limited education services

Caregivers stated a lack of information can make it difficult to care for a loved one: ‘Until I understood that dementia was what she was struggling with, I would get frustrated and angry with her and she would get the same with me. But as soon as I realised she wasn't doing stuff deliberately, that just made a world of difference’ (P8). Many caregivers wanted more education on the different types of dementia, stages, and how to deal with different behaviours: ‘I think it would've helped… if I’d known what it was… If someone had said ‘look they all go on like this and they say all these things’, then I probably wouldn't have tried to argue it out with him’ (P3). One tangible recommendation to increase knowledge on dementia was to provide a documentary: ‘…have a program like a documentary with people of different types of dementia and different stages, so that that can help highlight for people, you know, give them insights into maybe this is what our family member is experiencing or I am experiencing’ (P8). Other specific recommendations included more information on Power of Attorney, dealing with finances, and the latest research. Caregivers also felt that the wider community need to be better educated about dementia: ‘The wider community need to be educated. They're frightened’ (P17). Similarly, several caregivers felt health professionals need to be better educated about dementia and available services: ‘I don't really think GP’s know a lot about it either, what's happening or what's available’ (P6).

### 3.3 Facilitators of service use

#### 3.3.1 Empathetic

Caregivers noted the organisation helped them deal with difficult emotions by understanding and acknowledging their experiences: ‘It's good to have someone (Community Educator) tell you it's okay and to feel what you need to feel’ (P13). The organisation also provided support by normalising and legitimising feelings: ‘She (CE) made it quite clear that that's actually a normal reaction for someone’ (P5). Because of their privacy needs, caregivers appreciated the strong and trusting relationships they had with the Community Educator: ‘… you can talk about anything… Somebody (Community Educator) that understands and it's private, you're not burdening anybody with it’ (P10). Similarly, caregivers noted that they didn't feel guilty expressing emotions with the Community Educator: ‘And it's a place where you can cry and not feel guilty… She's just such a lovely person and when you're down and out, that's what you need’ (P10). Others mentioned the Community Educator formed a strong relationship with the PWD and this helped to overcome privacy concerns: ‘… she (Community Educator) has been invaluable because she can be there without being intrusive… she's safe, that's what I told her, she's a safe person’ (P17). Some caregivers mentioned that the Community Educator was the only person the PWD related to: ‘… when my wife was here, she (Community Educator) was one of the few people from the various agencies that she probably related to’ (P5). One caregiver described the relationship with the Community Educator as similar to that with a midwife: ‘The fact that she's made the effort to build a relationship with me, that's huge… I likened it to having my babies, because I had a midwife… I just need that one person; I don't need to spill my guts to everybody. I can spill it to her and that's enough’ (P9).

#### 3.3.2 Support groups

Support groups helped caregivers feel understood: ‘You met people who were in the same position as you’ (P6) and reduced feelings of seclusion: ‘It's good to know you aren't on your own [support group] and you have support’ (P7). Caregivers also noted that a benefit of the support groups was that they were private: ‘whatever you said in the meeting didn't go out of the meeting’ (P6).

#### 3.3.3 Proactive regular contact

The Community Educator helped overcome seclusion by keeping in touch with clients: ‘She keeps in touch … I told her at the beginning that I didn't need anything at the time but I’d really like the contact’ (P2). Often this contact was on a regular basis: ‘And she rings me at least once a month’ (P19). Caregivers noted they valued the support from the Community Educator because they often did not want to socialise with others: ‘Because there is a lot of grief and in those times, you're not very hospitable or social but yet you still need people. And that's where [CE] has been invaluable…’ (P17). The proactive service provided by the Community Educators broke through the seclusion: ‘…she always calls, and she initiates the call so I don't have to do the ringing… I like that, that she rings to see how things are going’ (P9). The regular contact meant that caregivers could share their concerns: ‘[I] often put off phoning people and every time [CE] rings there's always something I run past her or bounce off her and I usually say to her I’ve been thinking of ringing for the past 10 days, thank you for calling… because you do put it off’ (P17). Others mentioned they were grateful that Community Educator insists on visiting if they sense a problem: ‘I got teary and she said I’m back on the 13th and I’m coming out to see you whether you like it or not’ (P10).

#### 3.3.4 Provision of information/education/referrals

The knowledge provided by the Community Educator was noted to be invaluable: ‘Each time I’ve rung her up (CE) she's had a solution or a direction to send me in… So, once again she came to the rescue’ (P17). Caregivers also mentioned the organisations written resources were excellent: ‘I found [the organisation] excellent in that they provided me with resources’ (P8). Caregivers appreciated the knowledge the organisation provided regarding transitioning a PWD into care: ‘The Alzheimers Association provides a whole heap of information on the logistics, particularly about the care and what's involved and transitions from home to residency’ (P5). It was also noted that the organisation points caregivers in the right direction even if they do not have the resources themselves: ‘I’ve been able to phone [the organisation] and it was a bit outside their remit, but they were able to point me in the right direction, so they've become a trusted resource for me’ (P8).

## 4 DISCUSSION

This study addresses the recommendation by Stephan et al. (2018) and Queluz et al. (2020) to unpack caregiver needs. We do this by providing in‐depth insight into caregivers’ perceptions of barriers and facilitators of service use from one Alzheimers organisation. Many identified barriers concur with past research, however, novel to the current study is uncovering how an organisation overcomes barriers. Specifically, through *empathetic staff*, providing *support groups*, *proactively contacting clients* and *providing expert information and education*. These findings are imperative to inform community dementia support services how to overcome service use barriers. The findings also provide insight into caregivers’ perceptions of services provided by an Alzheimers Association; findings that are pertinent given Alzheimers Associations are provided worldwide (Wortmann, 2012) and argued to be an essential service in dementia care (Brodaty & Donkin, 2009), despite a dearth of research demonstrating value to clients.

Like past research, the current findings revealed emotions to be a prominent caregiver concern, and barrier, to service use (Queluz et al., 2020). Relevant emotions included, guilt (Innes et al., 2005) and grief, perceptions were that the organisation helped caregivers deal with difficult emotions through empathetic staff legitimising and acknowledging feelings and normalising their experiences and emotions. Previous research has argued for a need to normalise the use of services (Brodaty et al., 2005); our findings suggest it is equally important to normalise the difficult emotions caregivers’ experience. A desire for privacy (Stephan et al., 2018), feelings of pride (Lloyd & Stirling, 2011) and embarrassment were also barriers to requesting help. Caregivers reported that the empathetic Community Educators overcame these concerns by building strong and safe relationships with the caregiver and PWD.

A further barrier to service utilisation was caregiver's sense of burden and not wanting to be a nuisance to the organisation. Caregivers stated the organisation overcame this barrier by proactively contacting clients; because clients often struggled to ask for help. The findings exemplified that without the organisation being proactive, many of the caregivers would have continued to ‘try and cope’ on their own. The proactive contact provided by the Community Educator, also overcame the barrier that many caregivers become housebound (Granbo et al., 2019). Caregivers recommended that the organisation be more proactive by setting up regular planned catch‐ups with caregivers, improving on the current sporadic process. Caregivers also noted that a fear that others would not understand their situation prevented them seeking support. The organisation overcame these fears using empathetic staff and support groups, where caregivers had the opportunity to support, and be supported by others, in a similar situation (Chien et al., 2011; Lauritzen et al., 2015). The private nature of the support groups was also valued by caregivers.

Information/education provided by the organisation was a prominent facilitator of service use. Caregivers felt that you could never have enough information and a lack of information is a barrier to providing optimal care. Caregivers noted that the organisation met their information needs by providing invaluable knowledge, excellent written resources, and direction to other services. The specialist knowledge and skills were clearly valuable to overcoming the concerns of the PWD regarding service use. Several caregivers noted that the PWD was only comfortable with staff from the Alzheimers organisation being in their home. Caregivers did, however, recommend improvements including providing more education sessions on the stages and types of dementia, and more education and information to general practitioners, whom they felt lacked skills and knowledge around the resources and support offered by Alzheimers Associations and other organisations (Laparidou et al., 2019). Although the organisation does currently offer some education sessions for clients and the community, these are subsidiary to the primary role of the organisation and occur infrequently.

Importantly, two areas were identified as a weakness of the organisation. Similarly, to previous research, caregivers expressed a need for personalised or tailored services (Granbo et al., 2019) to enable the PWD to engage in meaningful activities (Black et al., 2013) that felt like hobbies and aligned with their interests. Caregivers recommended that the organisation draw on volunteers to help meet the individual needs of a PWD. Although the organisation provides some activities for the PWD, these are not funded by the government, and these occur on an infrequent basis. Furthermore, in concordance with previous research showing a lack of awareness of community services (Ploeg et al., 2009), caregivers felt the organisation needed to promote the organisation and their services more proactively.

The findings provide clear evidence of the value caregivers receive from the support of one Alzheimers organisation. The organisation provided essential emotional support, specialist knowledge and information, which overcame client's barriers to service use. The use of key empathic support workers, who formed strong safe trusting relationships with clients, and proactively contacted clients, ensured clients were supported and reduced isolation. Similarly, the support groups provided by the organisation also reduced seclusion. Despite others arguing that Alzheimers associations provide emotional support (Brodaty & Donkin, 2009), this is the first study to demonstrate client's perceptions of the value received. Moreover, the overarching theme in this study, that the experience of caregiving, and a caregiver's ability to cope, is different for everyone, exemplifies the necessity of specialist support people being able to provide individualised care. Based on the findings of this study, and in line with the wishes of clients to be involved in improving services (Granbo et al., 2019) we have developed six key recommendations for dementia service providers.

### 4.1 Recommendations for service providers

- Provide key support workers who can form strong relationships with clients, demonstrate empathy, show understanding and normalise and legitimise emotions.
- Be proactive and arrange regular scheduled meetings with clients to overcome client reluctance to ask for help.
- Provide support groups to facilitate social interaction amongst clients experiencing similar situations.
- Ensure the organisation has specialist knowledge and makes this knowledge accessible to clients through a variety of channels (face‐to‐face; written resources; education sessions). Focus on available services; the stages and types of dementia; transitioning a PWD into care; financial and legal advice.
- Tailor services to ensure the PWD can engage in meaningful activities that resonate with their interests.
- Promote the organisation and the services provided to ensure health practitioners, the general public and clients are aware of services.

### 4.2 Limitations

The findings of this study must be interpreted considering the limitations. The small convenience sample, of predominantly females, were all clients of the Alzheimers organisation and had obviously overcome some barriers to service utilisation, thus limiting generalisability to other clients, non‐clients and other contexts. However, the fact that our findings on the barriers to service use resonated with international research suggests our sampling was sufficient. In addition, future research needs to partner with Māori to look at the service needs, facilitators and barriers of service use amongst Māori clients. Furthermore, we focused on service utilisation of one Alzheimers organisation. We call for future research to explore caregiver's perceived barriers and benefits of service use from Alzheimers Associations nationally and internationally. To provide greater understanding of the barriers to service use, we recommend future research focuses on caregivers who have declined to use services.

## 5 CONCLUSION

Past researchers have recommended that to overcome barriers to service use, organisations must provide tailored information about their services and the benefits of their services (Moholt et al., 2020); a recommendation that resonated with the current findings. However, our findings highlight that knowing about services is not enough to overcome the difficult emotions, which prevent caregivers from asking for help. Our findings emphasise the need for organisations to be proactive by regularly contacting clients, because clients put off contacting the organisation. This regular contact can also overcome feelings of seclusion. The findings also emphasise the importance of having a consistent, trusting, empathic support person to overcome the many difficult emotions caregivers experience. The current findings emphasising the important characteristics of a key support worker align with the findings on barriers to formal support service use (e.g. day care services) and solidify the findings by showing that organisational characteristics, previously identified largely by healthcare professionals (Stephan et al., 2018) are also pertinent to caregivers. Of importance, the findings also show that an organisation's ability to demonstrate empathy and understanding is of little use unless the organisation is proactive with contacting clients to facilitate service use in the first place. Finally, the study provides the first in‐depth understanding of the important and crucial role an Alzheimers Association can play in supporting a PWD and their caregiver, namely, by reducing seclusion, providing essential emotional support and critical expert information and knowledge.

## CONFLICT OF INTERESTS

Associate Professor Kirsten Robertson and Professor Maree Thyne are on the Board of Alzheimers Otago.

## AUTHOR CONTRIBUTIONS

Kirsten Robertson and Maree Thyne conceived and designed the study, interpreted the data, and authored and reviewed drafts of the paper. Ivanna Matheson, collected data, interpreted the data, and authored and reviewed drafts of the paper. All the authors approve of the version to be published.

## HUMAN ETHICS

This study was reviewed and approved by the University of Otago Human Ethics Committee [Reference 19/155].

## Data Availability

The data that support the findings of this study are available on request from the corresponding author. The data are not publicly available due to privacy or ethical restrictions.
